# Diagnosis of Nitrogen Nutrition in Rice Leaves Influenced by Potassium Levels

**DOI:** 10.3389/fpls.2020.00165

**Published:** 2020-02-27

**Authors:** Wenfeng Hou, Merle Tränkner, Jianwei Lu, Jinyao Yan, Siyuan Huang, Tao Ren, Rihuan Cong, Xiaokun Li

**Affiliations:** ^1^ Key Laboratory of Arable Land Conservation (Middle and Lower Reaches of Yangtze River), Ministry of Agriculture/Microelement Research Center/College of Resources and Environment, Huazhong Agricultural University, Wuhan, China; ^2^ Department of Crop Sciences, Institute of Applied Plant Nutrition (IAPN), Georg-August-University Göttingen, Göttingen, Germany

**Keywords:** chlorophyll, leaf greenness, nitrogen, potassium, SPAD

## Abstract

Evaluation of nitrogen (N) status by leaf color is a kind of classic nutritional diagnostic method. However, the color of leaves is influenced not only by N, but also by other nutrients such as potassium (K). Two-year field trials with a factorial combination of N and K were conducted to investigate the effects of different N and K rates on soil plant analysis development (SPAD) readings and leaf N, K, magnesium (Mg), and chlorophyll concentrations. Visual inspections in leaf greenness revealed darker green leaves with increasing N rates, while paler green leaves with increasing K rates. Data showed that SPAD readings, chlorophyll, N and Mg concentrations, and the chloroplast area increased significantly with raising N rates, while declined sharply with the increase in K rates due to the antagonistic relationships between K^+^ and NH_4_^+^ as well as Mg^2+^. It was also probable that the increase in K promoted the growth of leaves and diluted their N and Mg concentrations. The paler leaf appearance resulting from the application of K may overestimate the actual demand for N in the diagnosis of rice N status. The strong antagonistic relationships between K^+^, NH_4_^+^, and Mg^2+^ should be considered in rice production and fertilization.

## Introduction

Rice (*Oryza Sativa* L.) is one of the most important crops feeding the world's population (Chapagain and Hoekstra, 2011). Predicted results showed that 70% more food will be needed by 2050 (Long, 2012). With the increasing population and decreasing arable land, it is of great importance to ensure food safety by improving rice yield per unit area (Jiang et al., 2016). Nitrogen (N) and potassium (K) are two elements that are required in great quantities for rice growth and yield formation (Pettigrew, 2008; Zhu et al., 2008; Oosterhuis et al., 2014). However, there are several problems concerning the application of N and K fertilizers in paddy fields.

Farmers usually apply excessive amounts of N to ensure high grain yields, which greatly depend on N inputs (Castillejo et al., 2010; Kaufman et al., 2013). Although the supply of N significantly improves grain yield, the low N use efficiency for the surplus application of N has been a major characteristic of rice production systems (Zhang et al., 2009; Qiao et al., 2012). In addition, the high input of N has substantial economic and environmental costs and results lodging (Guo et al., 2010; Zhang et al., 2016). In contrast, the importance of K has sometimes been over looked because the grain yield response to K is generally lower than that observed for N (Baligar, 2001). Farmers are also compelled to prioritize N over K due to the financial constraints (Hoa et al., 2006; Andrist-Rangel et al., 2007). The removal of crop residues from the field also accelerates the consumption of soil K (Timsina et al., 2013). As a result, low-K availability in paddy soils is a critical factor restricting the increase of grain yield (Zhang et al., 2013; Qiu et al., 2014; Zörb et al., 2014).

Leaves are the principal organs responsible for photosynthesis in vascular plants and contribute more than 90% of the crop biomass (Makino, 2011; Xiong et al., 2016). The supply of N could delay leaf senescence, maintaining the photosynthetic activity and rate for a longer period (Luo et al., 2018). Nitrogen plays important roles in leaf photosynthesis and the formation of grain yield, because it is required for the synthesis of numerous cellular components, such as amino acids, proteins, chlorophyll, and nucleic acids (Kusano et al., 2011; Makino, 2011; Pradhan et al., 2014). Approximately, 80% of leaf N is allocated to the chloroplasts and approximately 50% of such N is in the form of photosynthetic proteins, including those important for the light harvesting process, electron transport, and the enzymatic machinery of carbon metabolism (Xiong et al., 2015). Nitrogen deficiency could significantly decrease the N concentration, leaf area and chlorophyll concentration, SPAD readings, photosynthesis, and lead to lower biomass (Zhao et al., 2005; Berenguer et al., 2009). Potassium is not a functional or structural component within organic molecules in plants but functions as an inorganic cation incell extension, stomatal movement, osmoregulation, enzyme activation, photosynthesis, protein synthesis, phloem loading, and transport (Pettigrew, 2008; Zörb et al., 2014). Furthermore, K deficiency restricts the diffusion of CO_2_ through the leaf mesophyll and results in low substrate availability for carbon (C) fixation in chloroplasts (Jákli et al., 2016; Hou et al., 2018). These two factors, the chlorophyll concentration and restricted CO_2_ diffusion through the mesophyll, may contribute to a reduction in photosynthesis.

Previous studies have shown highly positive correlations between SPAD (Soil Plant Analysis Development) readings and chlorophyll and N concentrations (Singh et al., 2002; Wang et al., 2014; Ata-Ul-Karim et al., 2016). The use of SPAD measurements has been widely applied to monitor rice N status and to evaluate N demand of rice at different growth stages for improving grain yield and N use efficiency (Khurana et al., 2007; Huang et al., 2008). In a field trial we observed that there were obvious differences in leaf greenness between different K rates at low N rates, whereas no obvious differences in leaf greenness between different K rates at high N rates.

Thereby, in relation to this observation, the objectives of this work were: (i) to investigate the effects of different N and K rates on leaf greenness and (ii) to evaluate the effects of different levels of leaf greenness on grain yield.

## Materials and Methods

### Experimental Site and Experimental Design

**Figure 1:**
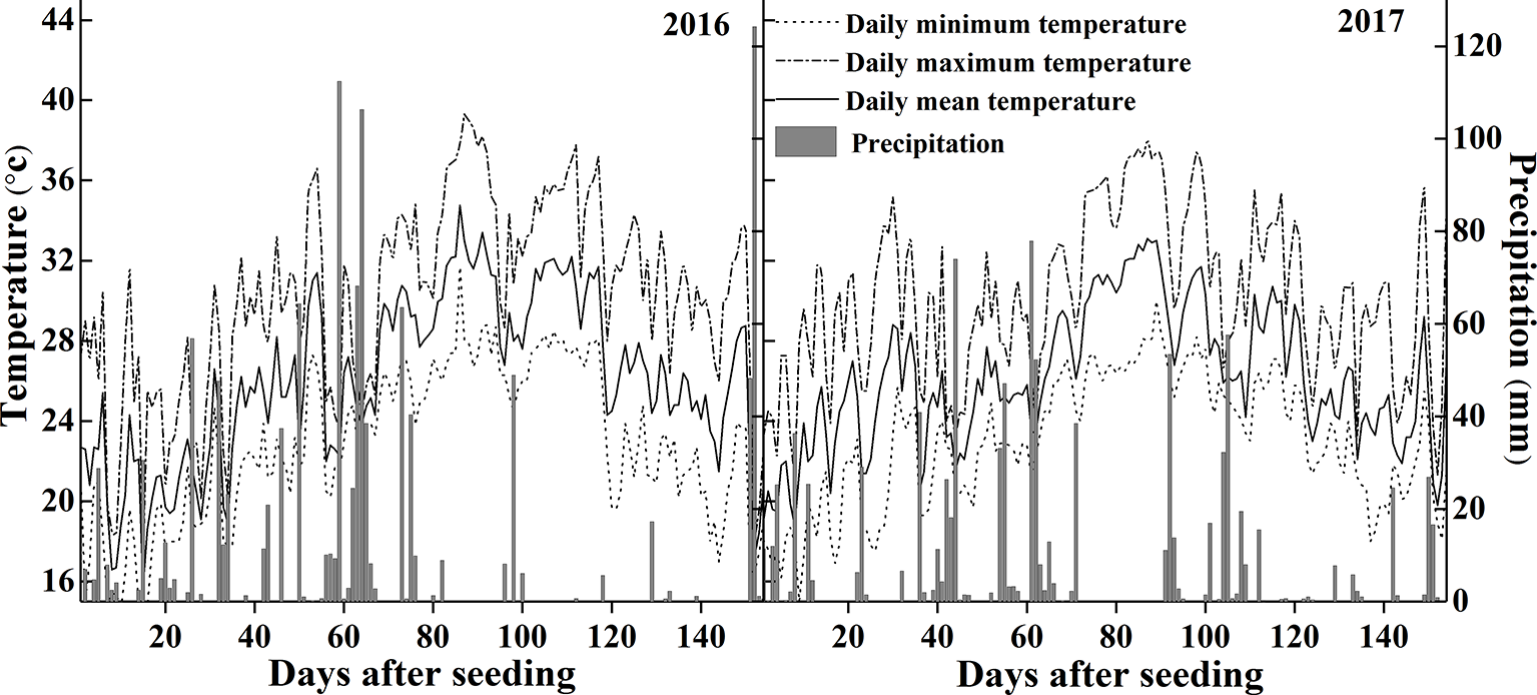
Temperature and precipitation during the rice growing seasons in 2016 and 2017.

The experiments were conducted in Wuxue County (30°06'46”N, 115°36'9”E), Hubei province, central China in 2016 and 2017. **Figure 1** shows the temperature and precipitation within the rice growing seasons of 2016 and 2017. The daily mean temperature was 24.3°C and 24.9°C, the daily mean precipitation was 11.3 mm and 6.7 mm respectively. Plants were grown in a sandy loam field with 16.4% clay, 36.8% silt, 46.8% sand, a pH of 5.8 (soil: water = 1: 2.5), organic matter concentration of 32.1 g kg^−1^, total N of 1.8 g kg^−1^, Olsen-P of 13.4 mg kg^−1^, readily available K of 44.5 mg kg^−1^, and slowly available K of 302.5 mg kg^−1^.

The field experiment was designed as a completely randomized block experiment with four N (main plots) and four K (subplots) fertilizer rates. The N treatments were: 0 kg N ha^−1^ (N0), 90 kg N ha^−1^ (N90), 180 kg N ha^−1^ (N180), and 270 kg N ha^−1^ (N270). The K treatments were: 0 kg K_2_O ha^−1^ (K0), 60 kg K_2_O ha^−1^ (K60), 120 kg K_2_O ha^−1^ (K120), and 180 kg K_2_O ha^−1^ (K180). All treatments supplied with 90 kg P_2_O_5_ ha^−1^. Urea (46% N), superphosphate (12% P_2_O_5_), and K chloride (60% K_2_O) supplied as the N, P, and K fertilizer sources, respectively. The N fertilizer was applied in three doses; 50% of the N fertilizer was applied as basal fertilizer (1 day before transplanting), and another 50% was divided into two equal parts and applied as top-dressing at the tillering stage (8 days after transplanting) and filling stage (56 days after transplanting). The K fertilizer applied in two doses: 75% as basal fertilizer and 25% at the filling stage. The P fertilizer applied as basal fertilizer. All plots plowed and leveled after the application of basal fertilizer. The plot area was 20 m^2^ (4 m × 5 m). All possible combinations of the N and K fertilization levels were present in the experiment, resulting in a total number of 16 individual treatments, and each of them was repeated three times.

### Crop Cultivation

Seeds of *Oryza sativa* L. ssp. *Japonica* (Shenliangyou 5814) were sown on May 22 and 20 and transplanted on June 29 and 22 in 2016 and 2017, respectively, after the application of basal fertilizer with a hill space of 0.24 m × 0.15 m. All plots were separated by soil bunds, which were covered with plastic film to prevent the exchange of water and fertilizer between neighboring plots. All treatments received the same fungicide, insecticide, and herbicide treatments, and no major diseases, pests, or weeds were present during the rice growing seasons. The plants were harvested on October 1, 2016, and September 30, 2017, respectively.

### SPAD Reading

A chlorophyll meter (SPAD-502, Minolta Camera Co., Osaka, Japan) was used for the SPAD reading measurements of the uppermost fully expanded leaves of each plot at the stage of stem elongation (65 days after sowing). Six expanded leaves were selected from each plot. Three SPAD readings per leaf were taken around the midpoint of the lamina and 30 mm away on both sides of the midpoint. The SPAD reading of each plot was obtained from the average of 18 SPAD readings (Peng et al., 1993). Sampled the leaf immediately after the measurement of SPAD readings, and used to analysis the parameters as described below.

After the test of SPAD, an aerial photograph was taken by an unmanned aerial vehicle (Phantom 4PRO V2.0, DJI-Innovations, Shenzhen, China) with a flight altitude of 13 m.

### Morpho-Physiological Analysis: Chlorophyll Concentration, Nitrogen Concentration, Potassium Concentration, and Magnesium Concentration

Leaf discs were obtained by a hole punch with a radius of 0.25 mm (the midvein was avoided) at stem elongation (65 days after sowing). Some of the leaf discs were used to measure the chlorophyll concentrations according to the method of Arnon (1949). Placed the other part of the leaf discs in an oven for 30 min at 105°C to stop metabolic activities and then dried at 70°C to a constant weight. All dried samples were ground, then digested by H_2_SO_4_ (purity of 98%) and H_2_O_2_ (purity of 30%) (for the analysis of N and K) on an open digestion furnace (LWY84B, Sichuan, China) with a temperature of 380°C and extracted with 4 M HNO_3_ (purity of 68%) for 4 h (for the analysis of Mg) (Thomas et al., 1967; Neuhaus et al., 2013). The N concentration in the digestion solution was determined by a continuous flow analysis (AA3, Seal Analytical Inc., Southampton, UK). The K concentration in the digestion solution was determined by a flame photometer (M-410, Sherwood Scientific Ltd., Cambridge, UK). The Mg concentration in the extract solution was determined by an atomic absorption spectrometer (Fast Sequential Atomic Absorption Spectrometer, AAS, AA240FS, Varian, California, USA).

### Anatomical Analysis: Leaf Thickness, Chloroplast Number, and Chloroplast Size

Anatomical analysis was down in 2017. Leaf segments (approximately 1 mm× 4 mm) were cut from the middle of the leaves (the midvein was avoided) using a blade at stem elongation (65 days after sowing), fixed immediately in 2.5% glutaraldehyde (v/v) in 0.1 mol L^−1^ phosphate buffer (pH 7.4), and then post fixed with 2% osmium tetroxide for 2 h. Leaf segments were embedded in Spurr's epoxy resin (Sigma-Aldrich, St. Louis, USA). Semithin leaf cross sections were stained with toluidine blue and observed at 200× magnification with a light microscope (IX71, Olympus Optical, Tokyo, Japan). Soft Imaging System Software (version 1.15.3, Panoramic Viewer, 3DHISTECH, Budapest, Hungary) was used for the observation and photography. Leaf thickness was measured using Image-Pro Plus (version 6.0, Media Cybernetics, Maryland, USA).

Ultrathin leaf sections (90 nm thick) were examined for the ultrastructural observation of chloroplasts using a transmission electron microscope (H-7650, Hitachi, Japan) after staining the leaf sections with 2% uranyl acetate (w/v) and lead citrate. Chloroplast number was counted, and the chloroplast length and thickness were measured using Image-Pro Plus. The chloroplast surface area was calculated according to the Cesaro formula:

$$S=4\times \pi \times \sqrt[{3}]{{\left(L\times T^{2}\right)}^{2}}$$

where L = 0.5× chloroplast length, and T = 0.5× chloroplast thickness.

### Determination of Total Leaf Area, Total Leaf Dry Mass, Specific Leaf Weight (SLW), Total Chlorophyll Concentration, and Grain Yield

Six plants were sampled at stem elongation (65 days after sowing). All green leaves were removed from the stem, and the single leaf area was calculated as single leaf area = leaf length × leaf width × 0.75 (Wang et al., 2007). The leaves were placed in an oven for 30 min at 105°C to stop metabolic activities and then oven dried at 70°C to a constant weight. Two hundred dry leaf discs from section 2.4 were weighed, and the SLW was calculated as SLW = the weight of 200 leaf discs/the area of 200 leaf discs. The area of the leaf discs = π × *r* ^2^, where *r* is 2.5 mm. The total chlorophyll concentration was calculated based on the chlorophyll concentration of the leaves and the total leaf area.

Grain yield was determined from an area of 10 m^2^ in the center of each plot on October 1, 2016 and September 30, 2017. Grain moisture concentration was measured using a moisture meter and adjusted to 0.14 g H_2_O g^−1^ fresh weight.

### Statistical Analyses

The data are expressed as the means ± standard deviation. Analysis of variance (*ANOVA*) is performed using SPSS 18.0 (SPSS, Chicago, IL, USA) to determine the significant effects of the N and K treatments on the described parameters. The least significant difference (LSD) test is used to compare significant differences based on *P*-values ≤ 0.05. All the figures and regression analyses was performed using Origin Pro 8.5 software (Origin Lab Corporation, Northampton, MA, USA).

## Results

**Figure 2:**
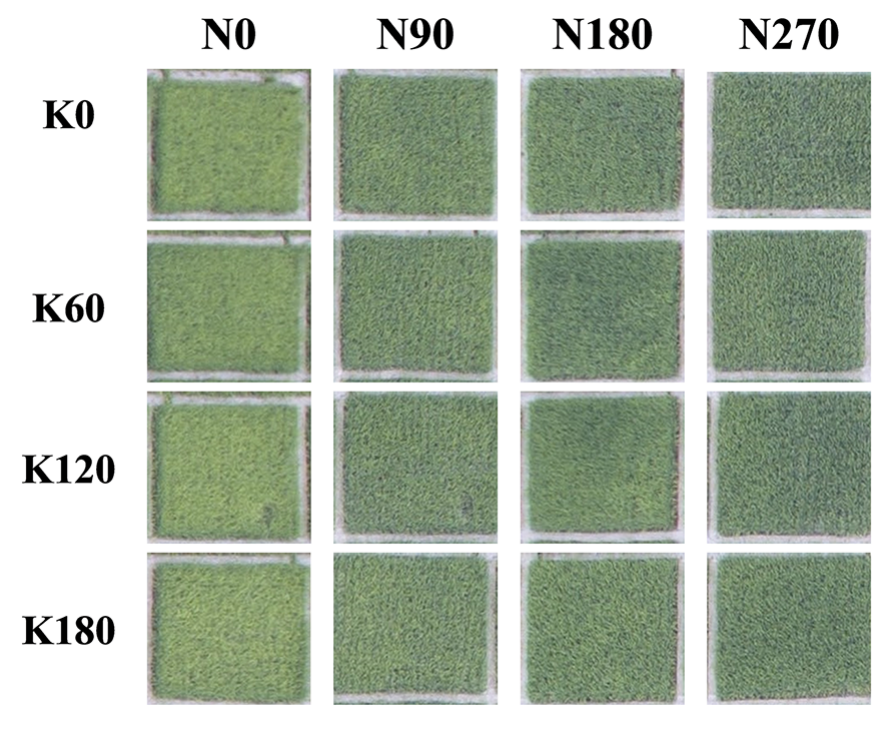
Differences in rice leaf color of different N and K fertilizer rates. Pictures were taken an unmanned aerial vehicle with a flight attitude of 13 m at 65 days after transplantation in 2016.

**Figure 2** shows the phenomenon in the field that how the rice leaf greenness varied with differences in N and K rates. Leaf greenness increased with increasing N and decreased with increasing K. Several physiological and anatomical properties were studied in this paper to explain this phenomenon.

### Leaf SPAD Reading and Chlorophyll Concentration

**Figure 3:**
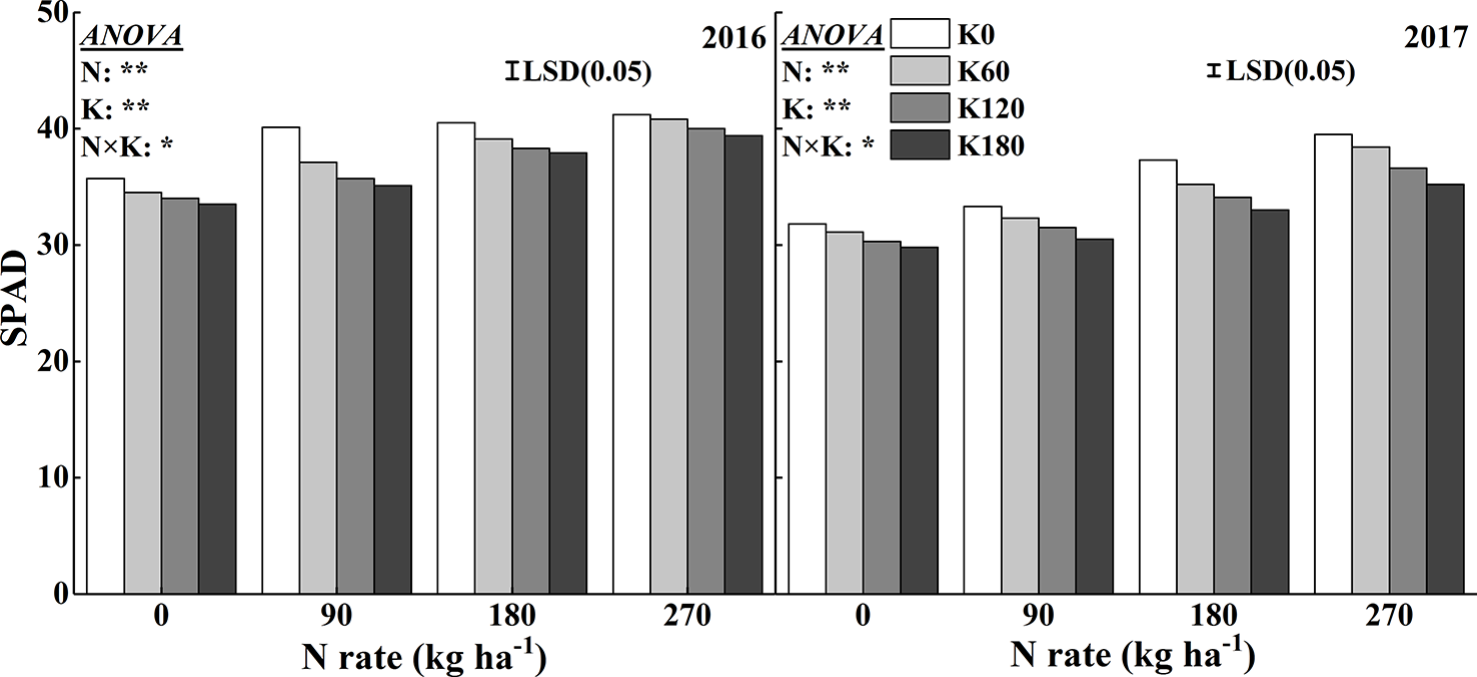
Effects of N and K rates on SPAD of rice leaves. LSD (0.05) represents the least significant difference among the treatments at *P* < 0.05.

The SPAD reading and chlorophyll concentration were used to quantify the leaf greenness. Increasing N fertilizer rates significantly increased the SPAD reading (**Figure 3**). The highest mean SPAD reading reached at 270 N and 0 kg ha^−1^. Compared to the N0 treatment, the application of 270 kg N ha^−1^ increased the SPAD readings by 15.3%, 18.1%, 17.4%, and 17.7% in 2016 and by 24.3%, 23.5%, 21.0, and 18.4% in 2017, respectively, at the four K rates. Within one N rate, increasing K application decreased the SPAD reading. The lowest mean SPAD reading reached at 0 N and 180 K kg ha^−1^. Compared to the K0 treatment, the application of 180 kg K_2_O ha^−1^ decreased the SPAD reading by 6.3%, 12.5%, 6.4%, and 4.3% in 2016 and by 6.4%, 8.4%, 11.5%, and 10.7% in 2017, respectively, at the four N rates.

**Figure 4:**
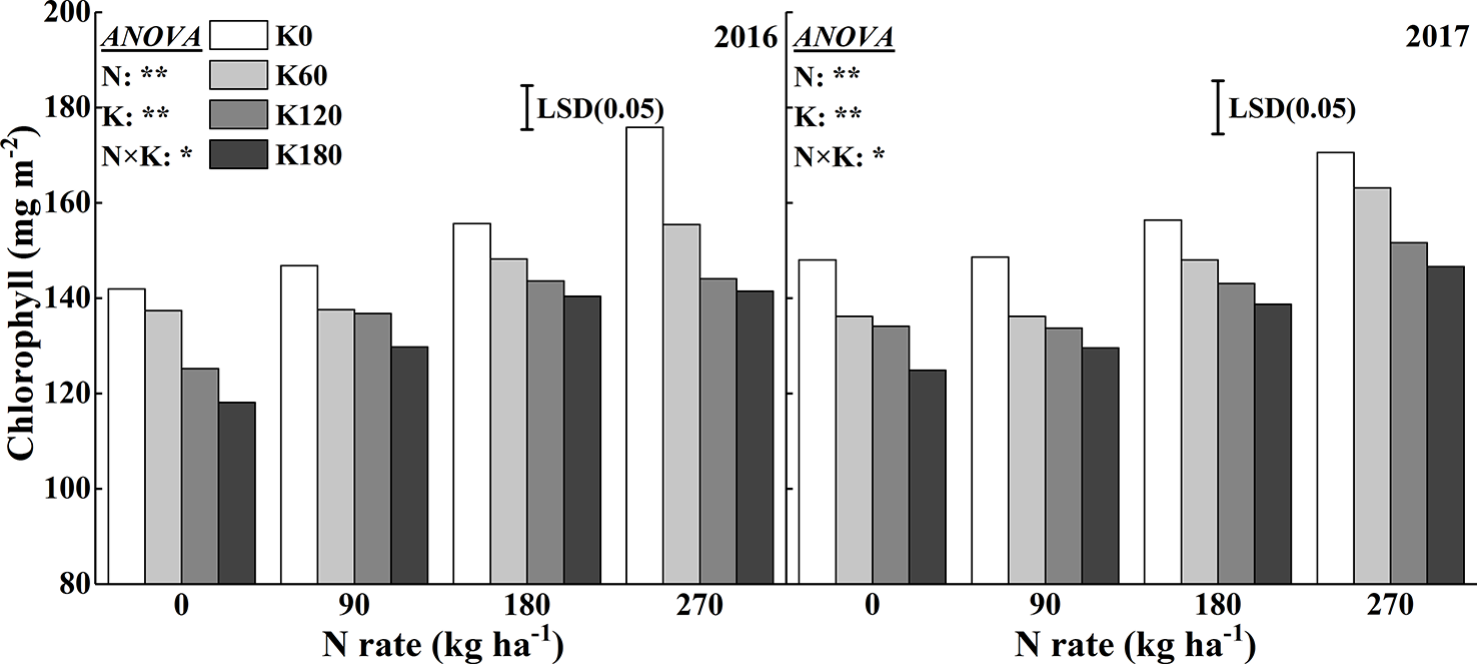
Effects of N and K rates on the chlorophyll concentration of rice leaves. Least significant difference (LSD) (0.05) represents the least significant difference among the treatments at *P* < 0.05.

Similar to the SPAD reading, the chlorophyll concentration significantly increased with increasing N rates (**Figure 4**). Compared to the N0 treatment, the application of 270 kg N ha^−1^ increased the chlorophyll concentration by 19.7%, 13.1%, 15.1%, and 19.8% in 2016 and 17.9%, 19.8%, 16.0%, and 20.6% in 2017, respectively, at the four K rates. Within a N rate, increasing the K rates decreased the chlorophyll concentrations. Compared to the K0 treatment, the application of 180 kg K_2_O ha^−1^ decreased the chlorophyll concentration by 19.6%, 8.5%, 9.8%, and 19.5% in 2016 and by 16.0%, 12.8%, 13.1%, and 14.1% in 2017, respectively, at the four N rates.

### Leaf N, K, and Mg Concentration

**Figure 5:**
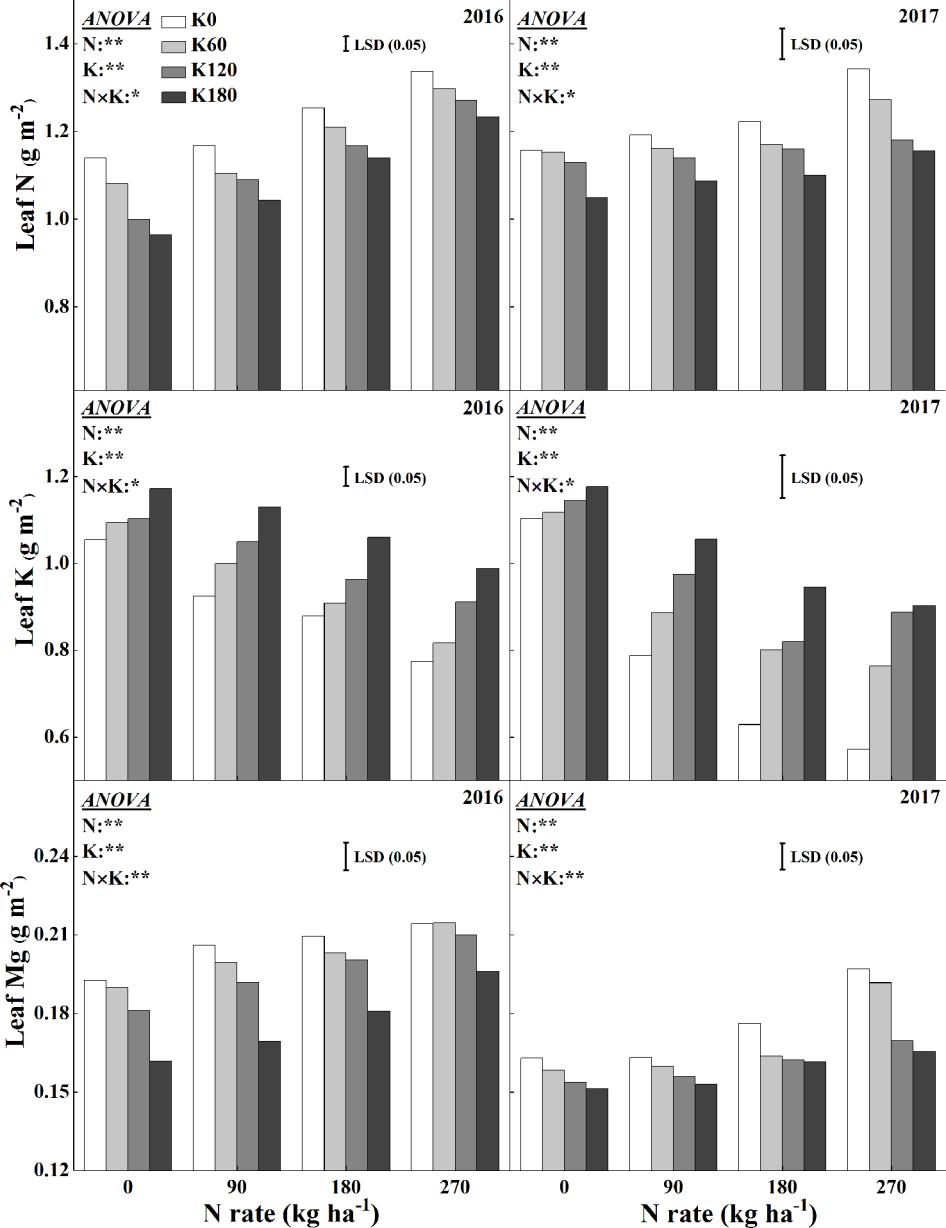
Effects of N and K rates on leaf N, K, and Mg concentrations. Least significant difference (LSD) (0.05) represents the least significant difference among the treatments at *P* < 0.05.

**Figure 5** shows the variations in leaf N, K, and Mg concentrations for the combined use of N and K. There were significant interaction effects of N and K on leaf N, K, and Mg concentrations. The concentration of N in leaves consistently increased as more N applied, while it decreased as more K applied. Compared to the N0 treatment, the application of 270 kg N ha^−1^ increased the N concentration by 46.6%, 44.8%, 47.8%, and 46.0% in 2016 and 32.1%, 32.3%, 35.4%, and 30.8% in 2017, respectively, at the four K rates. Within the same N rate, increasing K rate decreased the leaf N concentration. The lowest mean N concentration was reached at 0 N and 180 K kg ha^−1^. Compared to the K0 treatment, the application of 180 kg K_2_O ha^−1^ decreased the leaf N concentration by 6.3%, 12.6%, 11.5%, and 6.7% in 2016 and by 12.7%, 11.4%, 14.6%, and 13.6% in 2017, respectively, at the four N rates. The Mg concentration showed the same trend as that of the leaf N concentration. Compared to the N0 treatment, the application of 270 kg N ha^−1^ increased the Mg concentration by 18.1%, 7.0%, 6.0%, and 5.5% in 2016 and by 47.5%, 49.4%, 31.5%, and 19.3% in 2017, respectively, at the four K rates. Within the same N rate, increasing K rate also decreased the leaf Mg concentration. The lowest mean Mg concentration was reached for the combined use of 0 kg N ha^−1^ and 180 kg K_2_O ha^−1^. Compared to the K0 treatment, the application of 180 kg K_2_O ha^−1^ decreased the leaf Mg concentration by 16.0%, 12.3%, 10.7%, and 25.0% in 2016 and 9.5%, 10.6%, 15.1%, and 26.8% in 2017, respectively, at the four N rates.

### Chloroplast Development

**Figure 6:**
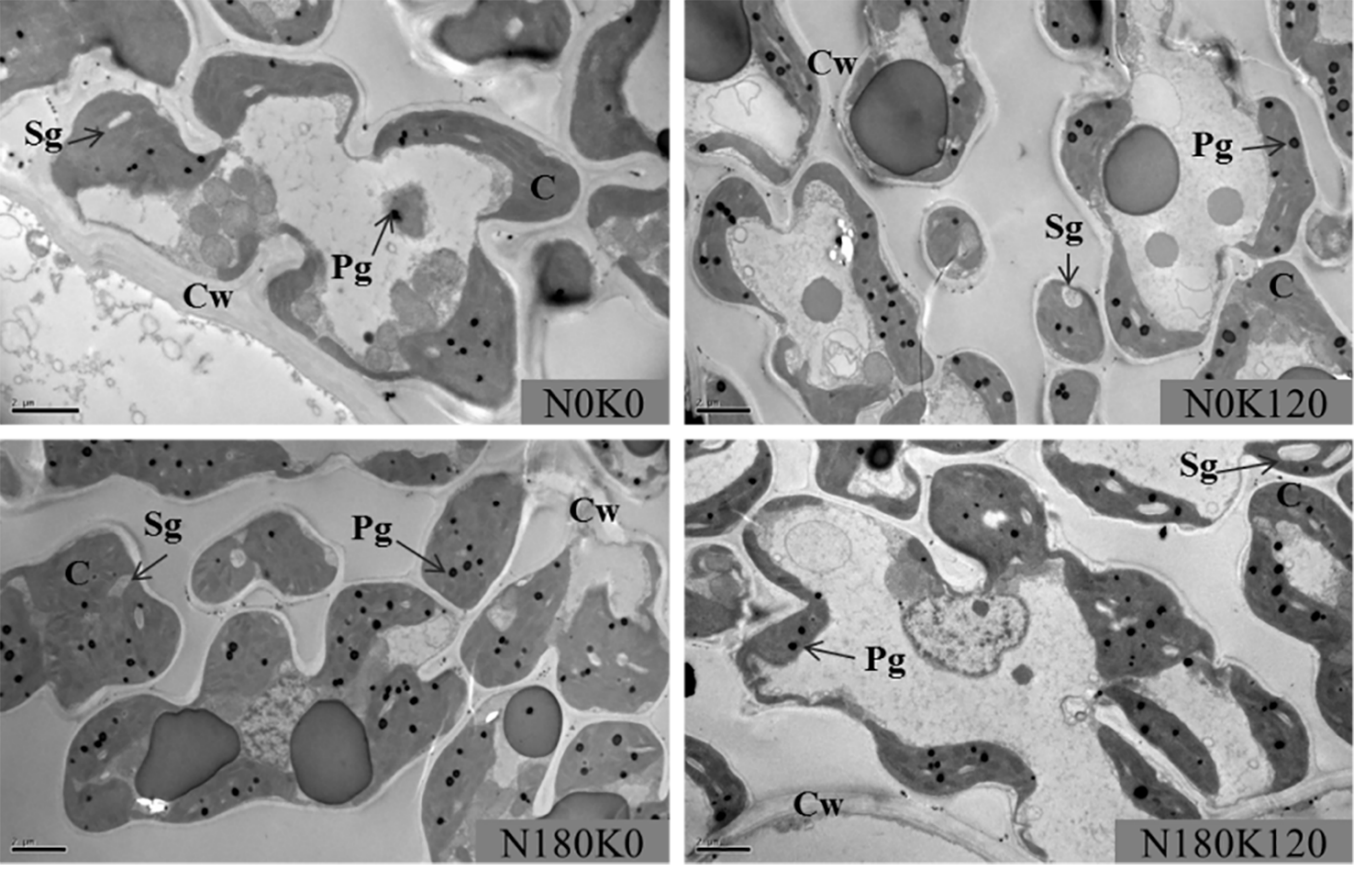
Transmission electron microscopy images of leaves plants fertilized with different N and K rates. C, chloroplast; Cw, cell wall; Pg, plastoglobuli; Sg, starch grain. The scale bar depicts 2 µm.

**Figure 7:**
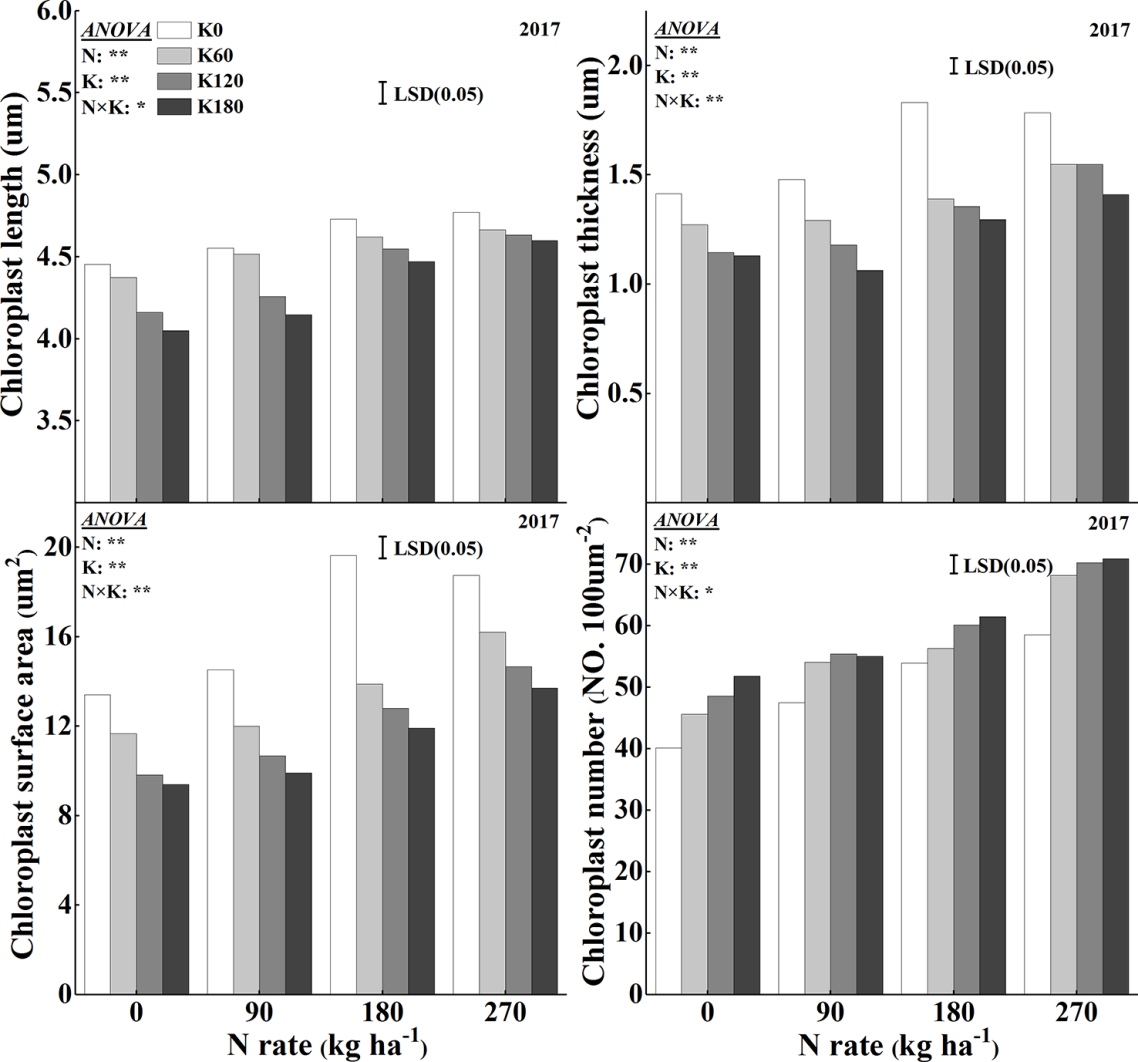
Effects of N and K rates on length, thickness, surface area, and number of chloroplasts. Least significant difference (LSD) (0.05) represents the least significant difference among the treatments at *P* < 0.05.

The chloroplast ultrastructure was significantly influenced for the combined supply of N and K (**Figures 6** and **7**). The application of N significantly enlarged the chloroplast, while the application of K significantly changed the chloroplast shape from an irregular large oval to a regular spindle shapes (**Figure 6**). Another characteristic was that the deficiency in K led to swollen chloroplasts, which were irregularly arranged in the cells. The application of N increased chloroplast length, thickness, and single chloroplast surface area, while the application of K decreased all such parameters (**Figure 7**). Chloroplast length, chloroplast thickness, and single chloroplast surface area were increased by 2.6–9.8%, 3.4–25.0%, and 6.4–43.0%, respectively, when plants were fertilized with 270 kg N ha^−1^ compared with N0 treatment. The chloroplast length, thickness, and single chloroplast surface area decreased by 1.5–6.7%, 13.8–22.7%, and 18.9–32.2%, respectively, when the K rate increased from 0 to 180 kg K_2_O ha^−1^ within the same N rate. The application of N and K both significantly increased the chloroplast number per unit leaf area. The chloroplast number per unit leaf area was increased by 13.2–44.0% and 11.4–19.6% with the application of N and K, respectively.

### Leaf Thickness and SLW

**Figure 8:**
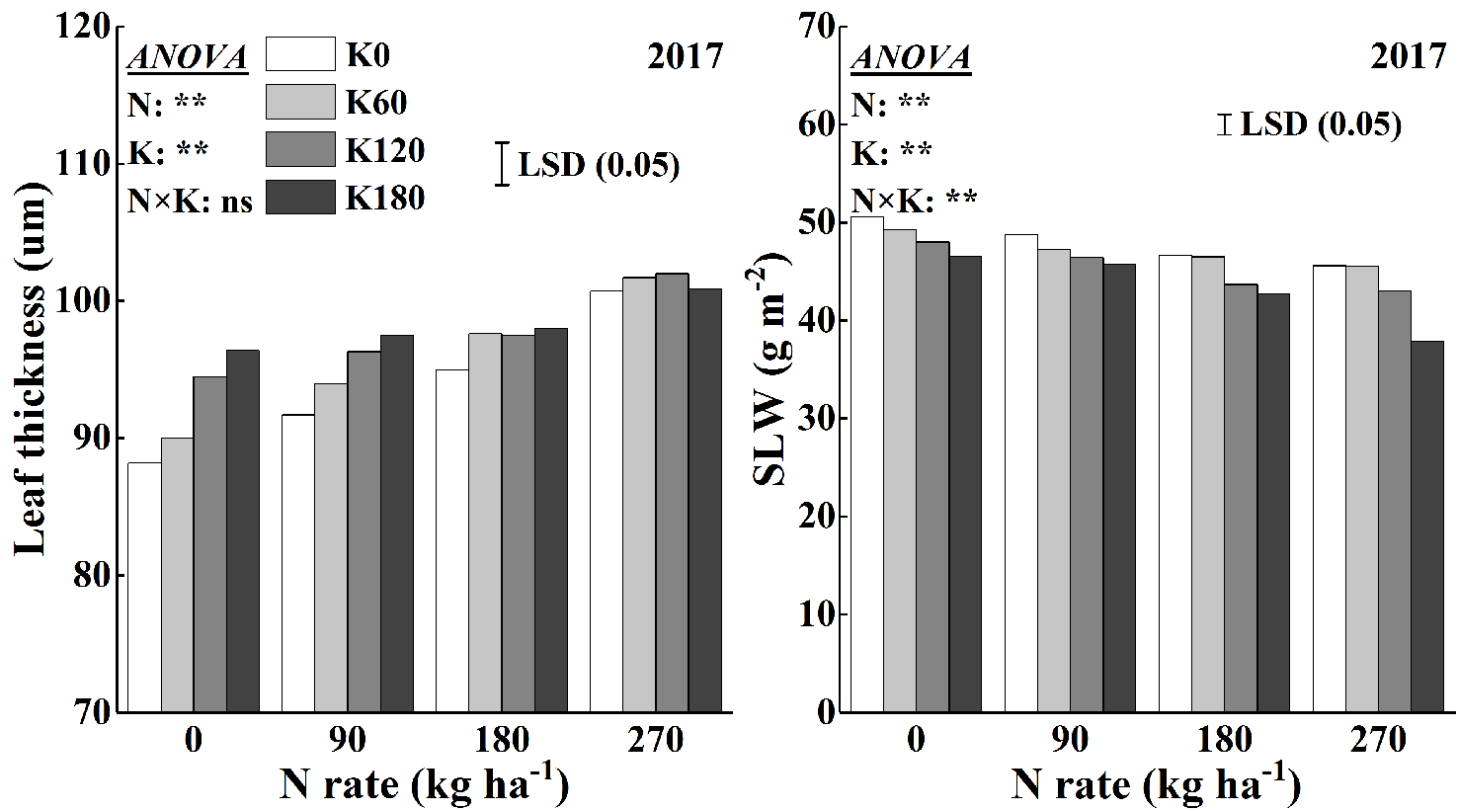
Effects of N and K rates on leaf thickness and specific leaf weight (SLW). Least significant difference (LSD) (0.05) represents the least significant difference among the treatments at *P* < 0.05.

Leaf thickness and SLW were analyzed to reflect the effects of N and K on leaf anatomy (**Figure 8**). The application of N and K increased leaf thickness. Nitrogen application increased leaf thickness by 2.8–9.8% compared to that of the N0 treatment, and within N rate, K application increased leaf thickness by 2.0–4.6%. The greatest leaf thickness reached at an application rate of 270 kg N ha^−1^, but within this treatment, K application had no significant effect. The SLW was significantly influenced by the application of N and K. N application decreased the SLW, and within N rate, K application decreased the SLW. The SLW was less sensitive to K rates than to N rates, as a significant decrease in SLW was observed only at the highest K application rate of 180 kg K_2_O ha^−1^. The SLW decreased on average by 2.9–11.1% and 2.3–9.9%, respectively, for the application of N and K compared to that for the N0 and K0 treatments. The lowest SLW observed at the highest fertilizer rates (270 kg N ha^−1^ combined with 180 kg K_2_O ha^−1^).

### Leaf Development and Grain Yield

The increase in N and K rates not only increased the leaf area index (LAI), but also increased the leaf dry mass and chlorophyll accumulation (**Table 1**). The lowest LAI, leaf dry mass, and chlorophyll accumulation values were observed in rice plants without N and K. Compared to N0K0 treatment, the maximum increase rate of LAI, leaf dry mass, and chlorophyll accumulation was 183.9%, 181.6%, and 178.7% in 2016 and 156.1%, 181.6%, and 156.1% in 2017, respectively. The application of N increased the LAI, the leaf dry mass, and chlorophyll accumulation by 60.5%, 73.2%, and 79.9% in 2016 and 71.5%, 111.2%, and 71.5% in 2017. The application of K increased these values by 28.2%, 18.8%, and 13.7% in 2016 and 25.8%, 18.7%, and 25.8% in 2017, respectively. There were significant interaction effects of N and K on LAI, leaf dry mass, and chlorophyll accumulation.

**Table 1:** Effects of nitrogen and potassium rates on leaf area index (LAI), leaf dry mass and chlorophyll accumulation.

Treatments		2016			2017		
		LAI	Leaf dry mass (g plant^−1^)	Chlorophyll accumulation (g plant^−1^)	LAI	Leaf dry mass (g plant^−1^)	Chlorophyll accumulation (g plant^−1^)
N0	K0	1.62 j	3.13 i	8.84 h	1.67 j	2.40 j	9.10 j
	K60	2.16 i	4.02 h	10.77 g	1.85 j	2.50 ij	10.08 j
	K120	2.32 hi	4.36 h	10.96 g	2.17 i	2.80 i	11.81 i
	K180	2.54 gh	4.86 g	11.12 g	2.27 hi	3.27 h	12.39 hi
N90	K0	2.45 h	5.04 fg	12.87 f	2.51 h	3.87 g	13.67 h
	K60	2.76 fg	5.41 f	14.06 ef	2.87 g	4.27 f	15.66 g
	K120	2.96 ef	5.85 e	15.04 e	3.08 efg	4.40 ef	16.76 efg
	K180	3.02 e	6.11 e	14.52 ef	3.24 de	4.73 e	17.64 de
N180	K0	3.01 ef	6.19 e	17.31 d	2.91 fg	5.10 d	15.85 fg
	K60	3.44 d	7.08 d	18.64 d	3.35 cd	5.87 c	18.27 cd
	K120	3.50 d	7.76 c	18.90 d	3.95 b	6.20 bc	21.51 b
	K180	4.19 b	8.03 bc	21.82 bc	4.07 ab	6.47 b	22.17 ab
N270	K0	3.29 d	7.86 bc	21.44 c	3.16 def	6.23 b	17.22 def
	K60	3.89 c	8.19 b	22.38 bc	3.60 c	6.37 b	19.63 c
	K120	4.61 a	8.80 a	24.60 a	3.94 b	7.30 a	21.49 b
	K180	4.48 a	8.69 a	23.46 ab	4.28 a	7.40 a	23.32 a
N		**	**	**	**	**	**
K		**	**	**	**	**	**
N×K		**	*	*	*	**	*

Different lowercase letters in the same column indicate significant differences between different treatments.* and ** indicate significance at P < 0.05 and P < 0.01.

**Figure 9:**
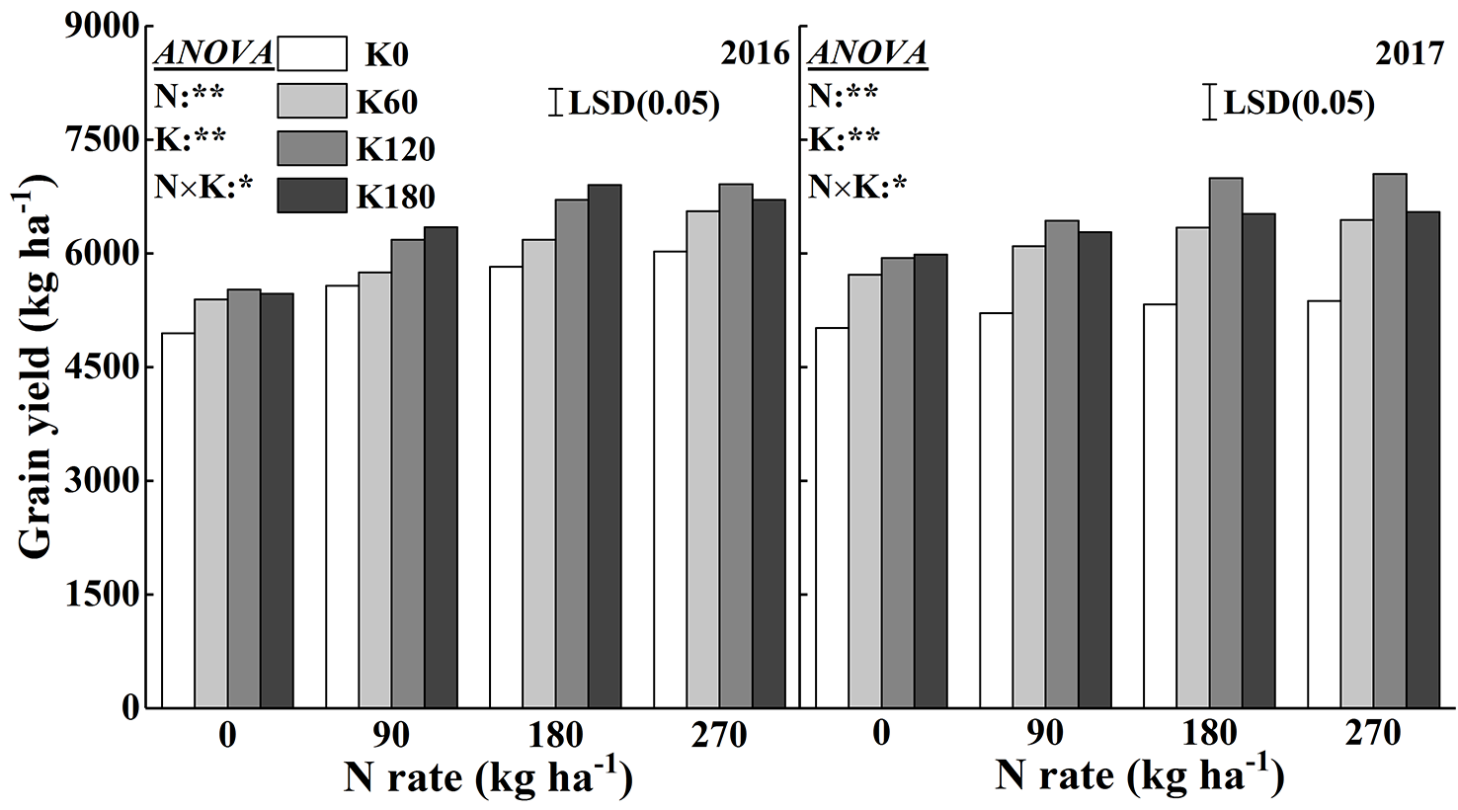
Effects of N and K rates on grain yield of rice in 2016 and 2017. Least significant difference (LSD) (0.05) represents the least significant difference among the treatments at *P* < 0.05.

**Figure 9** shows the variation in grain yields in response to the application of N and K. There were significant effects of N and K and their interaction effects on grain yield. Grain yield increased with increasing N and K rates. When compared to the yield from the N0 and K0 treatments, the grain yield increased on average by 11.8–22.8% and 6.7–13.7% in 2016 and by 17.5–21.4% and 6.0–12.1% in 2017, respectively, with the application of N and K. The combined use of 180 kg N ha^−1^ with 120 and 180 kg K_2_O ha^−1^ and 270 kg N ha^−1^ with 60, 120, and 180 kg K_2_O ha^−1^ achieved relatively high grain yields.

**Figure 10:**
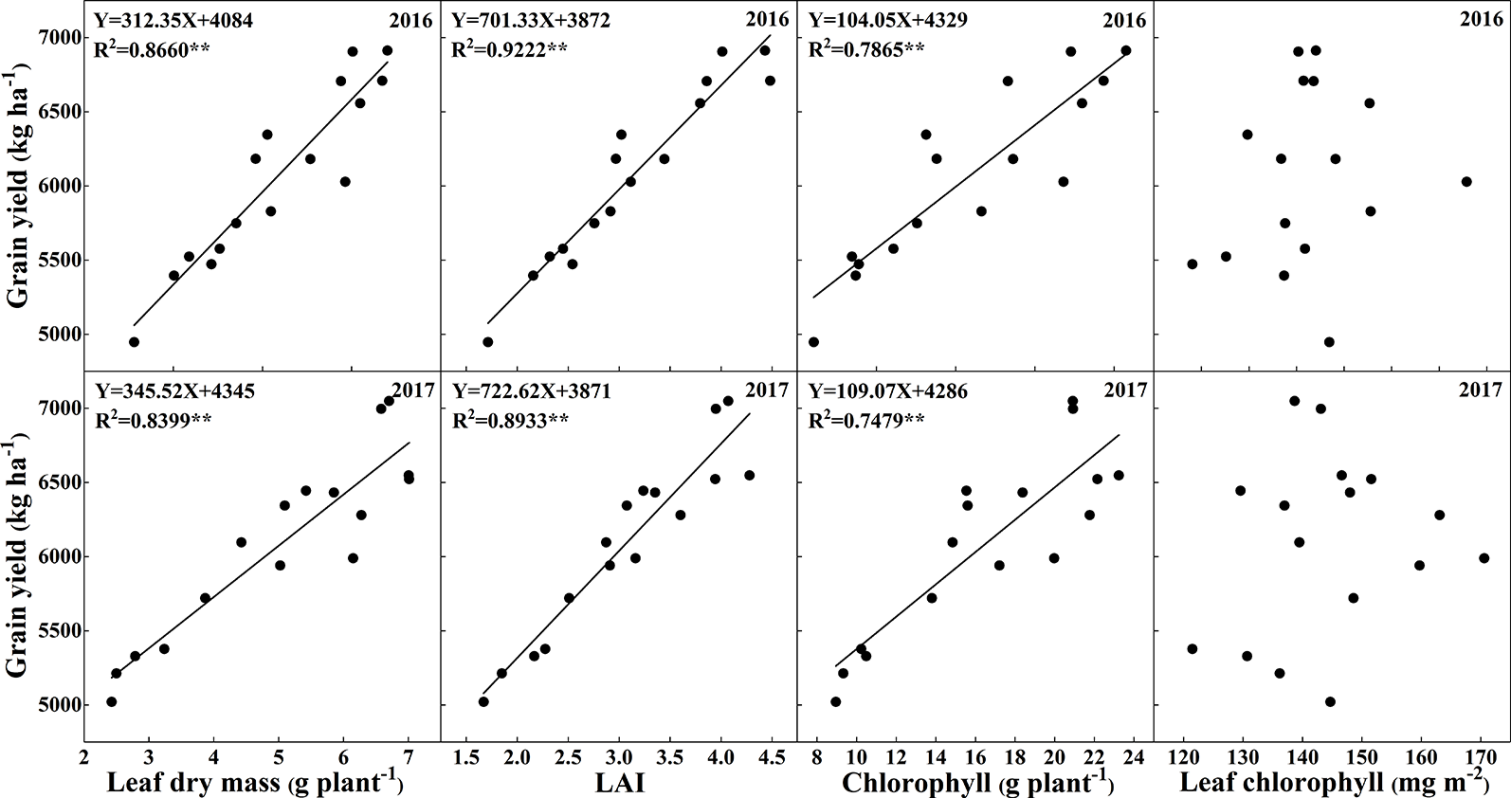
Relationships of total leaf dry mass, leaf area index, chlorophyll concentrations, and total chlorophyll accumulation rates with grain yield.

**Figure 10** shows the highly positive correlations between leaf dry mass, LAI, chlorophyll accumulation, and chlorophyll concentration with grain yield across the 2 years, which indicates that there were significant effects of leaf area, leaf dry mass and leaf chlorophyll accumulation on grain yield, and the chlorophyll concentration in this study did not show significant correlation with grain yield.

## Discussion

### Effects of Nitrogen and Potassium on Leaf Greenness

A visual inspection of leaf greenness revealed darker green colors with increasing N rates. Analyses of SPAD readings and chlorophyll concentrations could verify this visual observation. Many studies have investigated the relationships between leaf N concentrations and SPAD readings in the past 20 years, and it has been universally accepted that there were significant relationships between leaf N concentrations and SPAD readings as well as leaf chlorophyll concentrations (Castelli et al., 1996; Huang et al., 2008; Lavres and Monteiro, 2010; Wang et al., 2014). In addition to SPAD-502, visible and near infrared (VIS-NIR) hyperspectral imaging system, 18%-gray dark green color index (DGCI), and so on had been used to diagnose the leaf N status (Rotbart et al., 2013; Zhang et al., 2013; Confalonieri et al., 2015; Yuan et al., 2016). Numerous studies have shown that the application of N could increase chlorophyll concentrations (Li et al., 2009; Gu et al., 2017; Liu et al., 2017). However, there is considerable debate on whether the application of K could either increase or decrease the leaf chlorophyll concentrations. Studies on cotton and oilseed rape have shown that K application could increase chlorophyll concentrations (Zhao et al., 2001; Hu et al., 2016). Reduced leaf chlorophyll concentration under K deficiency has been reported in cotton, olive trees, and legumes (Collins and Duke, 1981; Zhao et al., 2001; Erel et al., 2015). In contrast, Jia et al. (2008) reported that leaf chlorophyll concentration increased at moderate K deficiency in rice. In the present study, increasing K rates decreased the SPAD readings and chlorophyll concentrations irrespective of the application of N. This phenomenon might be the result of the uptake antagonism between K^+^ and NH_4_^+^. Ammonia is the major N source in paddy fields, and NH_4_^+^ and K^+^ have similar hydrated diameters and charges, resulting in antagonistic relationships between these two ions (Zhang et al., 2010; Coskun et al., 2016).

Our results indicated that with the increase in K, leaf K concentrations increased while the N and Mg concentrations decreased. As both N and Mg are structural elements of chlorophyll, they have substantial effects on chlorophyll synthesis (Xiong et al., 2015; Ceylan et al., 2016; Tränkner et al., 2018). Approximately 50% of leaf N invested in the form of photosynthetic proteins, including for the important components in involved in light harvesting, such as chlorophylls (Xiong et al., 2015).

### Leaf Anatomical Characteristics on Leaf Greenness

The chloroplast number and size increased significantly with the N supplementation (Laza et al., 1993; Xiong et al., 2015). In line with this, in our results, the application of N increased chloroplast numbers per leaf area. However, the application of K could further increase the chloroplast number per leaf area, which is contrary to the results that K application decreased chlorophyll concentrations. Regarding the morphology of the chloroplasts, the application of K had different effects. Our data showed that chloroplast thickness and surface area decreased with the supplementation of K at a specific N rate, and that chloroplasts were swollen and irregularly arranged in the cells (**Figure 6**). The thinner chloroplasts under sufficient K application might be the result of proper thylakoid membrane stacking, which is a process that requires K ions to screen negative surface charges of the thylakoid membranes. Irregularly arranged thylakoids and swollen chloroplasts were observed in K-deficient cotton plants (Hu et al., 2016). Furthermore, the increased chloroplast thickness under the low N or K rates might be the result of the starch granules accumulation in the chloroplast (**Figure 6**). Large starch granules were observed in chloroplasts of N-deficient rice and K-deficient cotton and maize (Hall et al., 1972; Laza et al., 1993; Zhao et al., 2001; Li et al., 2013). The accumulation of starch inside the chloroplasts is most likely due to the lower supply of N, which weakens the demand for C compounds. It had been reported that the increase of N rates promoted the development of chloroplast and decreased the starch granules in chloroplast (Laza et al., 1993; Li et al., 2013; Gu et al., 2017). On the other hand, K deficiency significantly decreases the remobilization of carbohydrate, increases leaf carbohydrate concentrations, and starch granules (Jia et al., 2008; Gerardeaux et al., 2009; Wang et al., 2012). Sucrose and starch are the main products of photosynthesis, which provide carbon and energy for plant growth. Starch is a temporarily stored carbohydrate, which can be converted into source (Gandin et al., 2009). It had been reported that K deficiency lead to significantly higher hexose, sucrose, and starch concentrations (Hu et al., 2015; Hu et al., 2017). An accumulation of starch inside the chloroplasts might also be the reason for the increased SLW under low K application rates. According to Pettigrew (2008), a deficiency in K decreased the transport of photosynthetic assimilates out of leaves, which may lead to the increase in SLW. Tränkner et al. (2018) also reported that a deficiency in K restricted the phloem loading of sucrose resulting in an accumulation of sucrose in source tissues. Zhao et al. (2001) reported that K-deficient cotton leaves had less intercellular air space than that supplied with sufficient K. Sims et al. (1998) showed that increased N levels could improve leaf thickness by increasing the mesophyll cell layer with only small changes in total epidermal thickness. However, a higher N supply leads to a larger leaf area, which might be a reason for the decreases of SLW.

### Relationship Between Leaf Greenness and Grain Yield

The leaves of rice are responsible for capturing solar energy, so a larger leaf area could capture more light, which in turn is an important factor for the productivity of dry mass and the formation of grain yield. In the present study, the LAI and total leaf dry mass were significantly increased with the supply of N and K. Chlorophyll is one of the most important pigments in leaves, and it is capable of transforming sunlight into dry mass through photosynthesis (Xiong et al., 2015). Several studies have shown the high correlations between crop yields and leaf chlorophyll concentrations (Scharf et al., 2006; Anand and Byju, 2008; Rorie et al., 2011). Interestingly, the chlorophyll concentrations decreased with increasing K rates, while the grain yield increased with increasing K rates. What explains this phenomenon? Previous studies had shown that higher leaf mass was accompanied by lower leaf N concentration, which could be a dilution effect due to the leaf expansion and increasing carbon compounds (Bussotti et al., 2000; Mediavilla and Escudero, 2003). The results indicated that the increase of K rates significantly decreased the chlorophyll concentrations and chlorophyll accumulation (**Figure 4** and **Table 1**), while increased the leaf area and dry mass (**Table 1**). Although the increase of K rates decreased the chlorophyll concentrations per leaf area of a single leaf, the total chlorophyll accumulation rates increased with the increase of K rates (**Table 1**). Thus, the decreased chlorophyll concentrations could be counterbalanced by the larger leaf area and dry mass, which in turn resulted in higher grain yield, as indicated by the significant positive relationships among grain yield, LAI, and leaf dry mass (**Figure 10**). The results indicated that the application of N and K could both significantly improve the total chlorophyll accumulation rates, and positively correlated with the grain yield (**Figure 9**).

In summary, the observed field phenomenon that the increase in K rates decreased leaf greenness but increased final grain yield can be explained as follows. The increased K rates decreased leaf N and Mg concentrations, which are two important nutrients in the synthesis of chlorophyll, and led to the decreases of chlorophyll concentrations and paler leaves. The more K was supplied, the paler the leaf became, especially under low N conditions. The increase of K rates also decreased leaf N and Mg concentrations under high N conditions. However, the differences in leaf greenness between different K rates could not be recognized by the naked eye under relatively high N rates compared with that under low N conditions. Furthermore, our data also indicate that SPAD readings cannot be used as reliable estimate of the relative grain yield because the highest SPAD readings not always resulted in the highest yield. The highest yield achieved with a combined use of 180 N and 180 K kg ha^−1^, and further increase of N and SPAD readings did not increase the grain yield.

## Conclusions

Contrary to the conventional understanding, at least at the jointing stage, rice leaf color was enhanced with the increase of N rates and decreased with the increase of K rates. However, the increased K rates decreased leaf greenness but increased grain yield. We proposed two hypotheses: (i) the increase of K rates decreased leaf N and Mg concentrations due to the antagonistic relationships between K^+^ and NH_4_^+^ as well as Mg^2+^, or (ii) the increase of K rates promoted leaf development, which diluted the leaf N and Mg concentrations.

Based on the field observations and research data, our results clearly demonstrated the following conclusions. (1) The application of K in a N deficient soil will exacerbate the deficiency of leaf N and further decrease leaf greenness, which will overestimate the actual demand for N fertilizer in diagnosing rice N status and waste N fertilizer. (2) The increase of total leaf area and dry mass could counterbalance the negative effects of decreased leaf N and chlorophyll concentration for the supply of K. The slight leaf greenness caused by the increase of K has no negative effects on grain yield, but will overstate the recommended N fertilizer in rice leaf N diagnosis. Based on this study, the application of N and K fertilizer in rice production systems should be balanced due to the antagonistic relationship between K^+^ and NH_4_^+^, and Mg fertilizer should be considered due to the antagonistic relationships between K^+^ and Mg^2+^.

## Data Availability Statement

The raw data supporting the conclusions of this article will be made available by the authors, without undue reservation, to any qualified researcher.

## Author Contributions

JL, RC, TR and XL conceived the idea and led the study design. WH performed experiments with support by JY and SH. WH wrote the manuscript. MT and XL provided advice and edited the manuscript. All authors read and approved the final manuscript.

## Funding

This work was supported by the National Key Research and Development Program of China (2016YFD0200108), the National Natural Science Foundation of China (31872174), the Special Fund for Agro-scientific Research in the Public Interest (No. 201503123), and the Fundamental Research Funds for the Central Universities (2662017JC010).

## Conflict of Interest

The authors declare that the research was conducted in the absence of any commercial or financial relationships that could be construed as a potential conflict of interest.
